# Association between Levels of Serum Ferritin and Bone Mineral Density in Korean Premenopausal and Postmenopausal Women: KNHANES 2008–2010

**DOI:** 10.1371/journal.pone.0114972

**Published:** 2014-12-18

**Authors:** Seung Joo Chon, Yun Rak Choi, Yun Ho Roh, Bo Hyon Yun, SiHyun Cho, Young Sik Choi, Byung Seok Lee, Seok Kyo Seo

**Affiliations:** 1 Department of Obstetrics and Gynecology, Severance Hospital, Yonsei University College of Medicine, Seoul, Republic of Korea; 2 Department of Orthopaedic Surgery, Severance Hospital, Yonsei University College of Medicine, Seoul, Republic of Korea; 3 Department of Biostatistics Collaboration, Yonsei University College of Medicine, Seoul, Republic of Korea; 4 Department of Obstetrics and Gynecology, Gangnam Severance Hospital, Yonsei University College of Medicine, Seoul, Republic of Korea; 5 Institute of Women's Life Medical Science, Yonsei University College of Medicine, Seoul, Republic of Korea; Van Andel Institute, United States of America

## Abstract

**Background:**

As women go through menopause, serum estrogen decreases and ferritin increases. Decreased serum estrogen is well known to cause detrimental effects on bone health; however, data on the associations of serum ferritin with BMD before and after menopause are still lacking. Therefore, this study aimed to investigate the association between serum ferritin levels and BMD in premenopausal and postmenopausal Korean women.

**Methods:**

This study was performed using data from the 2008–2010 Korean National Health and Nutrition Examination Survey, including 7300 women (4229 premenopausal and 3071 postmenopausal). BMD was measured using dual X-ray absorptiometry at the femur and the lumbar spine, and serum ferritin levels were measured by chemiluminescent immunoassay.

**Results:**

Median serum ferritin levels in postmenopausal women were higher than those in premenopausal women despite the same age ranges. Serum ferritin levels were only significantly correlated with BMD on the lumbar spine (β = −0.189, *p*-value = 0.005) in premenopausal women after adjusting confounding factors. Additionally, BMD on the lumbar spine had tended to decrease as serum ferritin quartiles increase (P for trend = 0.035) in premenopausal women after adjusting confounding factors. On the other hand, there were no significant associations between serum ferritin levels and BMD on the total femur and, femur neck in premenopausal women, and BMD on the total femur, femur neck, and lumbar spine in postmenopausal women.

**Conclusion:**

Increased serum ferritin levels were significantly associated with BMD in premenopausal women, particularly on the lumbar spine, but not in postmenopausal women.

## Introduction

Ferritin is an essential component of the body, but many studies have stated that ferritin that exceeds the normal physiological range may potentially cause health problems in women. Excessive iron is toxic, causing cellular dysfunction mainly as a powerful catalyst for the generation of highly toxic free radicals, which can damage all molecular classes found *in vivo* [1]. Modifications in these molecules are considered to cause adverse effects on diverse aspects of health, most notably, decrease in bone mineral density (BMD), which must be carefully considered as mortality due to osteoporotic hip fractures alone is the same as that from breast cancer [2], [3].

Recently, there has been clinical evidence to support an association between serum ferritin level and bone parameters. Researchers have concluded that higher body iron stores are significantly associated with lower bone mass at various skeletal sites and cause an increased prevalence of osteoporosis and fractures, especially in women ≥45 years old [4]. However, menopausal status was not considered as a factor that could potentially have a great effect in determining the bone mass in women.

The reason why menopause should be considered while evaluating BMD on women is that by going through this physiological phenomenon, women eventually experience changes on two important growth-associated molecules in their bodies [5]: decrease in estrogen and increase in ferritin. Estrogen deficiency is well known to cause detrimental effects on bone health [6]. Additionally, serum ferritin increases by 2–3 times during this period due to the lack of a major mechanism of iron excretion [7], while menstrual blood decreases [8]. However, as far as we know, there has been little data presented suggesting an association between serum ferritin and the status of BMD in women based on menopausal status.

Therefore, in this study, we observed differences in BMD on the total femur, femur neck, and lumbar spine with respect to serum ferritin level, and attempted to determine whether levels of serum ferritin have associations with BMD in Korean premenopausal and postmenopausal women.

## Methods

### Study population

This study was performed using data from the Korean National Health and Nutrition Examination Survey (KNHANES) (2008–2010 data), specifically data from the KNHANES IV survey (2008 and 2009 data) and the KNHANES V survey (2010 data), all performed by the Korean Ministry of Health and Welfare. KNHANES IV and V were each conducted over 3 years (2007–2009 and 2010–2012, respectively) using a rolling sample survey that involved a complex, stratified, multistage, probability-cluster survey of a representative sample of the non-institutionalized civilian population in South Korea. Sampling units were randomly selected, with 23 households from each primary sampling unit and 200 randomly selected sampling units, yielding 4600 households in 2008, whereas 192 sampling units were randomly selected, with 20 households from each primary sampling unit, yielding 3840 households in 2009 and 2010. The survey was composed of three parts: a health interview survey, a health examination survey, and a nutrition survey. All surveys were conducted by trained interviewers. The interviewers were not provided with any prior information regarding the specific participants before performing the interviews. All participants were provided with written informed consents to participate in this survey, and we received the data in anonymized form. The study was carried out in accordance with the ethical standards of the Helsinki Declaration, and was approved by the Yonsei University Health System, Severance Hospital, Institutional Review Board (4-2013-0706).

We excluded male participants and individuals who were less than 20 years old, pregnant, had aspartate aminotransferase (AST) or alanine aminotransferase (ALT) levels greater than 100 IU/L, hemoglobin (Hb) levels less than 10 g/dL, creatinine levels greater than 1.4 mg/dl, white blood cell (WBC) counts greater than 10000Thous/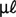 or less than 4000Thous/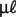 or ones who suffered from cardiovascular, liver, renal, or thyroid disease or cancer. In addition, participants were excluded if they had not provided blood samples for ferritin measurement or if their BMD on total femur, femur neck, and lumbar spine were not determined. People with serum ferritin levels less than 1.0 ng/mL or greater than 500.0 ng/mL were excluded to rule out those with possible ferritin-related disease such as hemochromatosis or sickle cell disease. Women who did not undergo anthropometric measurements of height and weight and ones who had undergone hysterectomy or bilateral salpingo-oophorectomy were excluded. The final sample consisted of 7,300 participants (4,229 premenopausal and 3,071 postmenopausal women).

### Variable measurements

Based on age, participants were grouped into 15 categories. Seoul (the capital city of Korea) and its surrounding metropolitan area (Gyonggi), along with six other cities (Incheon, Daejeon, Gwangju, Daegu, Busan, and Ulsan) were classified as urban areas, whereas the remaining regions in South Korea were classified as rural areas. Subjects were classified as current smokers (if they had smoked at least one cigarette per day during the previous 12 months), ever smokers, and never smokers. Alcohol consumption was evaluated and categorized into four groups, depending on history of drinking for the previous 12 months. Physical activity levels were divided into three categories, low, moderate, and high, according to the International Physical Activity Questionnaire short-form scoring protocol [9]. Education level was stratified into three categories: (1) elementary school (6 years of schooling); (2) middle, high school (7–12 years of schooling); and (3) college or university (>12 years of schooling). Occupation was classified into two categories: (1) people who work regularly in an office or indoors and (2) people who usually work outdoors and have rigorous physical activity. The circumstances of dietary intake were classified into four groups to predict whether participants had been supplied with sufficient nutrition (both quantity and quality) through their meals: (1) frequently insufficient quantity and quality, (2) occasionally insufficient quantity and quality, (3) sufficient quantity but poor quality, (4) sufficient quantity with diverse nutritional supplementations. Personal and family histories of bone fractures were surveyed, and HT usage was divided into two categories: ever and never users.

Anthropometric measurements were performed. Height and weight were measured with light clothing without shoes. Blood samples were collected early in the morning, after overnight fasting. Plasma concentrations of hemoglobin, vitamin D, ferritin, alkaline phosphatase (ALP), and parathyroid hormone (PTH) were measured following routine biochemical laboratory protocols. Ferritin was measured by chemiluminescent immunoassay with an ADVIA Centaur system (Siemens, USA) until February 15, 2008, and immunoradiometric assay with a 1470 WIZARD gamma counter (Perkin Elmer, Finland) from February 16, 2008 until 2010. All clinical analyses were performed at the Neodin Medical Institute, a laboratory certified by the Korean Ministry of Health and Welfare.

### Criteria and Definitions

Menopause is defined as amenorrhea for 12 months following the final menstrual period [10]. In our study, postmenopausal status was defined as the self-reported cessation of menstruation for more than 1 year only because we had already excluded women who had undergone hysterectomy or bilateral salpingo-oophorectomy. Among postmenopausal women, women less than 40 years old were excluded since they were not considered as physiologically menopaused women. Although serum ferritin ranges can vary among laboratories, levels exceeding 500.0 ng/mL were considered as a marker of iron overload disorders [11]. Values of serum ferritin were divided according to quartiles for each group of premenopausal and postmenopausal women. Osteoporosis is a systemic skeletal disease characterized by low bone density and micro-architectural deterioration of bone tissue with a consequent increase in bone fragility [12], diagnosed primarily based on BMD. According to a WHO Study Group, the diagnosis of osteoporosis among postmenopausal women is based on T-score thresholds. T-scores at or above -1.0 are considered normal, those between −1.0 and −2.5 as osteopenia, and those at or below −2.5 as osteoporosis [13].

### Statistical analysis

To analyze baseline characteristics according to menopausal status, data were expressed as means (± standard deviation; SD) for continuous variables after independent-sample *t* test and as percentages for categorical variables after chi square test, unless otherwise stated. One-way analysis of variance (ANOVA) was used to express graphs comparing serum ferritin values according to age groups depending on menopausal status. In order to compare BMD values on total femur, femur neck and lumbar spine according to serum ferritin quartiles after adjustment for confounding variables, analysis of covariance (ANCOVA) was used in premenopausal and postmenopausal women separately. We used simple linear logistic regression model to determine the independent effects of serum ferritin on BMD at various skeletal sites among premenopausal and postmenopausal Korean women. The covariates for the adjusted calculation were age, body mass index (BMI), smoking, drinking, exercise, serum vitamin D, and calcium and phosphate supplementation. Data analysis was carried out using SPSS software (version 20; SPSS, Chicago, IL), and *p*-values less than 0.05 were considered significant.

## Results

Table 1 shows baseline characteristics of premenopausal and postmenopausal women. BMI, serum ferritin, and vitamin D were statistically significantly higher in postmenopausal women than in premenopausal women, whereas calcium and phosphate supplementations and BMD on total femur, femur neck, and lumbar spine were lower in postmenopausal women than in premenopausal women (*p*-value<0.001).

**Table 1:** Baseline characteristics of premenopausal and postmenopausal women.

	Premenopause (n = 4229)	Postmenopause (n = 3071)	*p*-value
Age (years)	36.45±8.50	63.63±9.22	<0.001
Height (cm)	159.35±5.57	153.02±5.84	<0.001
Weight (kg)	57.40±9.19	56.68±8.58	0.001
BMI (kg/m^2^)	22.61±3.44	24.17±3.20	<0.001
Hb (g/dL)	12.83±1.18	13.11±0.97	<0.001
Serum Ferritin (ng/mL)	32.22±27.64	68.22±47.70	<0.001
Serum Vitamin D (ng/mL)	16.44±5.80	19.10±7.27	<0.001
Serum ALP (IU/L)	183.24±52.85	253.36±74.48	<0.001
Serum PTH (pg/mL)	66.67±23.49	68.13±29.87	0.446
Ca intake/day (mg)	467.06±300.56	419.17±392.40	<0.001
P intake/day (mg)	1042.90±435.36	945.38±430.67	<0.001
Total femur BMD (g/cm^2^)	0.90±0.11	0.77±0.12	<0.001
Femur neck BMD (g/cm^2^)	0.76±0.10	0.62±0.11	<0.001
Lumbar spine BMD (g/cm^2^)	0.99±0.12	0.80±0.14	<0.001
Resident district			<0.001
Urban	2968 (70.2)	179 (58.4)	
Rural	1261 (29.8)	1279 (41.6)	
Education			<0.001
Elementary	186 (4.4)	2039 (67.0)	
Middle, High school	2301 (54.6)	880 (28.9)	
University, College	1729 (41.0)	126 (4.1)	
Occupation			<0.001
Office type	3851 (91.5)	2178 (71.6)	
Outfield type	360 (8.5)	866 (28.4)	
Alcohol			<0.001
Never	2163 (51.3)	2523 (82.4)	
≤Once/month	1578 (37.4)	418 (13.7)	
>Once/month, ≤Once/week,	395 (9.4)	85 (2.8)	
>Once/week, ≤Everyday	84 (2.0)	35 (1.1)	
Smoking			<0.001
Never	3638 (88.5)	2790 (92.4)	
Ever	193 (4.7)	95 (3.1)	
Current	280 (6.8)	136 (4.5)	
Physical activity			0.001
Low	2684 (63.7)	2071 (67.9)	
Moderate	890 (21.1)	600 (19.7)	
High	640 (15.2)	381 (12.5)	
Dietary intake			<0.001
Frequently difficult	14 (0.4)	57 (2.0)	
Occasionally difficult	107 (2.8)	239 (8.5)	
Enough, but not diverse	1932 (50.3)	1569 (55.5)	
Diverse, Enough	1790 (46.6)	960 (34.0)	
Personal history of fracture			<0.001
Yes	101 (2.4)	637 (20.7)	
Family history of fracture			0.797
Yes	723 (17.1)	518 (16.9)	
HT			<0.001
No	4138 (97.8)	2637 (85.9)	
Yes	91 (2.2)	434 (14.1)	

BMI: Body mass index, Hb: Hemoglobin, ALP: Alkaline phosphatase, PTH: Parathyroid hormone, Ca: Calcium, P: Phosphate, BMD: Bone mineral density, HT: Hormonal therapy.

Reference range: Hb 12.0–16.0 g/dL; Ferritin 10.0–130.0 ng/mL; Vitamin D 30.0–100.0 ng/mL; ALP 39.0–117.0 IU/L; PTH 10.0–57.0 pg/mL.

**Figure 1:**
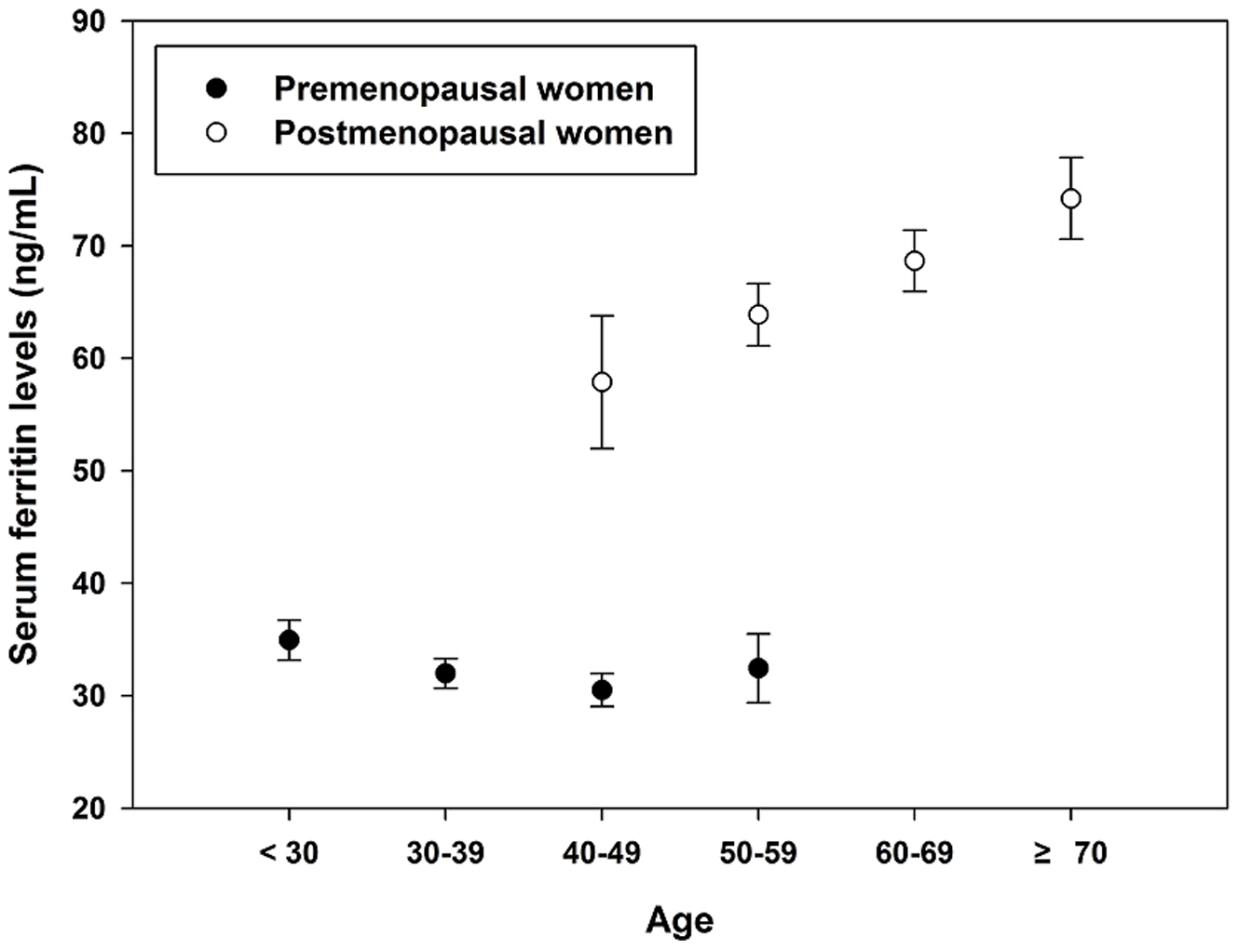
Median serum ferritin concentrations according to age group. As women become older, the median serum ferritin levels showed tendency to increase. After dividing women into two groups depending on menopausal status, the median serum ferritin in premenopausal women was found to be much lower than that in postmenopausal women, in between 40–59 years old.

Fig. 1 shows median serum ferritin levels according to age groups depending on menopausal status. As women became older, the median serum ferritin levels showed tendency to increase. After dividing women into two groups depending on menopausal status, the median serum ferritin in premenopausal women was found to be much lower than that in postmenopausal women, which was particularly notable in the overlapping age groups, between 40–59 years old.

To determine the associations in between serum ferritin and BMD at various skeletal sites, simple linear logistic regression analysis was performed after adjusting for age, BMI, smoking, drinking, exercise, serum hydroxyvitamin D levels, calcium and phosphate supplementations, personal and family histories of bone fractures. In premenopausal women, BMD on total femur and femur neck were not associated with differences in serum ferritin, whereas statistically significant decrease in BMD on lumbar spine was noted as serum ferritin increased (β = −0.189, *p*-value = 0.005). On the other hand, no statistically significant correlations were found in serum ferritin and BMD at any parts of skeletal sites in postmenopausal women (Table 2).

**Table 2:** Logistic regression analysis to determine the effects of serum ferritin on bone mineral density at various skeletal sites.

	Premenopause (n = 4229)			Postmenopause (n = 3071)		
Variables	β	SE(β)	*p*-value	β	SE(β)	*p*-value
Total femur (g/cm^2^)	−0.046	0.059	0.439	0.008	0.036	0.823
Femur neck (g/cm^2^)	−0.044	0.059	0.456	0.007	0.034	0.840
Lumbar spine (g/cm^2^)	−0.189	0.068	0.005	0.063	0.047	0.185

Adjusted variables: age, BMI, smoking, drinking, exercise, serum 25-hydroxyvitamin D levels, calcium and phosphate supplementations, personal history of fracture, family history of fracture.

**Figure 2:**
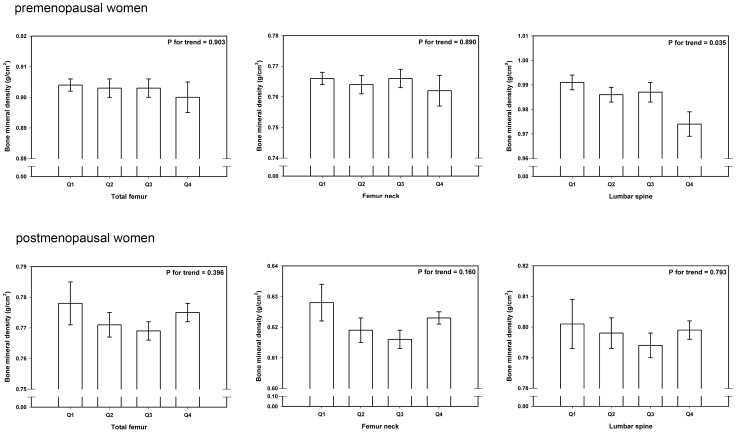
BMD according to serum ferritin quartiles after adjusting with confounders in premenopausal and postmenopausal women. Only lumbar spine in premenopausal women showed significantly decreased tendency in BMD as serum ferritin increased in quartiles (P for trend = 0.035). On the other hand, BMD on total femur and femur neck (P for trend = 0.903, 0.890, respectively) in premenopausal women. BMD on total femur, femur neck, and lumbar spine in postmenopausal women were not found to be statistically significantly associated (P for trend = 0.396, 0.160, 0.793, respectively).

After categorizing pre and postmenopausal women into four groups according to numbers of people having various serum ferritin concentrations [0.680–19.110 ng/mL for Q1, 19.120–37.690 ng/mL for Q2, 17.691–62.930 ng/mL for Q3, and 62.931–486.110 ng/mL for Q4], ANCOVA was used in order to investigate BMD at various skeletal sites according to serum ferritin quartiles after adjusting confounding variables. Only lumbar spine in premenopausal women showed significantly decreased tendency in BMD as serum ferritin increased in quartiles (P for trend = 0.035). On the other hand, BMD on total femur and femur neck (P for trend = 0.903, 0.890, respectively) in premenopausal women (Fig. 2), BMD on total femur, femur neck, and lumbar spine in postmenopausal women were not found to be statistically significantly associated (P for trend = 0.396, 0.160, 0.793, respectively) (Fig. 2).

## Discussion

We investigated the associations of serum ferritin with BMD based on menopausal status, which is a period when some people face detrimental effects on bone health. In the present study, an increase in serum ferritin was statistically significantly associated with a decrease in BMD on the lumbar spine; however, no statistically significant associations were found on the total femur and femur neck in premenopausal women. Conversely, no associations were found for serum ferritin with BMD on various skeletal sites in postmenopausal women.

Ferritin is a ubiquitous intracellular protein that provides stored iron in a non-toxic form, and ferritin levels reflect the amount of iron stored in the body. When levels of serum iron increase, the level of transferrin is not up-regulated, meaning that all iron molecules are not able to bind to transferrin. In an iron overloaded status, leftover iron that is not bound to transferrin may cause detrimental effects on tissues. Two main hypotheses attempt to explain this toxicity induced by iron overload: oxidative injury and lysosomal injury. The oxidative injury hypothesis proposes that iron overload *in vivo* results in the formation of oxyradicals, which damage cellular constituents including lipids, nucleic acids, proteins, and carbohydrates and impair calcium homeostasis and cellular function [14]. The lysosomal injury hypothesis proposes that excessive accumulation of iron within lysosomes can lead to lysosomal fragility, impairing lysosomal function through the release of hydrolytic enzymes and storing iron into the cytoplasm [14]. Progressive iron deposition can also result in cardiac, hepatic, and endocrine disorders [15], [16]. Endocrine dysfunction is a consequence of iron overload in hypothalamic-pituitary axis, and frequently manifests with abnormalities in pubertal development and menstrual irregularities in females [17].

A decrease of BMD has been considered to be a disease associated with abnormal calcium metabolism. Additionally, an increasing number of studies have strongly been suggesting the deleterious role of excessive iron on bone health [18]. In cases of hemochromatosis [19], African hemosiderosis [20], thalassemia [21], sickle cell disease [22], [23], and liver diseases [24], [25], iron overload could be suggested as a common mechanism causing bone loss. For example, in genetic hemochromatosis, osteopenia, and osteoporosis have been observed in 78.9% and 34.5% of patients with iron overloaded condition, respectively [26]. One previous study reported that sickle cell disease leads to many complications including osteoporosis and osteopenia, correlating especially strongly with increased ferritin level [27]. Likewise, overloaded iron acts in diverse ways, inhibiting osteoblast differentiation, stimulating osteoclast resorption, decreasing activity of alkaline phosphatase (ALP) which is an important enzyme in early osteogenesis [15], and also inhibiting anterior pituitary synthesis of gonadotropins [28]. On the other hand, there are also studies on contradictory results. In a previous animal study, dietary iron deficiency was also reported to affect bone negatively [29], and another report showed that osteoporosis patients are slightly iron deficient, suggesting that serum ferritin levels do not always have to be high to have detrimental effects on bone metabolism [30].

As previously mentioned, serum ferritins increase by 2–3 times during the menopausal period [31]. Given that overloaded serum ferritin, which is known to be a source of decrease in BMD, is more profound in postmenopausal women, we speculated that an increase in serum ferritin would cause more cases of osteoporosis in postmenopausal women. However, the outcome of our study was different from expected, with results presenting a statistically significant decrease on lumbar spine BMD as serum ferritin levels increased only in premenopausal women, whereas no significant associations were noted for serum ferritin with BMD of the total femur, femur neck and lumbar spine in postmenopausal women. Therefore, it is not serum ferritin which is important; rather it is the change in serum ferritin level that is important in interpreting associations with BMD, which we can also see from another previous large longitudinal study [32].

Postmenopause, associated with the loss of bone due to the anabolic and antiresorptive effects of ovarian hormones, has long been recognized as a risk for osteoporosis [33], [34]. The most rapid bone losses occur in menopausal transition and before menstrual cycles have completely stopped. Recently a number of studies have observed accelerated bone density loss in perimenopausal women prior to their menopause [35], [36], and longitudinal studies have also shown particularly high bone density loss during perimenopause and its continuation during the first few years of postmenopause [35], [37], [38]. In one previous study, higher estrogen levels were significantly correlated with less average bone density loss in premenopausal, perimenopausal and postmenopausal women [39]. For the first few years of menopause, serum ferritin levels do not seem to have an impact on BMD, but after a certain periods of time, increase in serum ferritin levels seem to affect decrease in BMD among postmenopausal women.

In our study, a statistically significant decrease in BMD was found only on the lumbar spine in premenopausal women. The lumbar spine is classified as trabecular bone, in which the mineralized bone matrices are rapidly lost because their large surface areas facilitate remodeling and their thin plates of mineralized matrix are easily perforated, resulting in the loss of trabecular connections [40], [41]. This high surface area-bone matrix volume configuration provides a large area facilitating the initiation of remodeling of the smaller mineralized trabecular bone matrix volume [39]. In a previous longitudinal population-based study, the rate of bone loss from the axial skeleton in women was found to be similar before and after 50 years of age, whereas bone loss at appendicular sites did not begin until midlife [42]. Also, in additional cross-sectional and longitudinal population-based studies, researchers confirmed that cortical volumetric BMD remained relatively stable until menopause and then decreased significantly, whereas trabecular bone loss began early, in the third decade and accelerated after menopause [43].

The age group of 45–55 years old is the most heterogeneous age group consisting of both premenopausal and postmenopausal women. Although in the same age group, premenopausal women have lower levels of serum ferritin with higher BMD, whereas postmenopausal women have higher levels of serum ferritin with lower BMD. Therefore, regardless of menopausal status, it is not appropriate to draw a conclusion, stating that women ≥45 years of age have serum ferritin levels which significantly associate with bone parameters [4], based on heterogeneous data.

The present study has some limitations. As this study is cross-sectional, direct associations between the variables of interest could not be clearly determined. Additionally, if we could have collected information on other factors that may have affected serum ferritin and BMD, we would have achieved more precise results. Furthermore, because we collected data retrospectively, we were unable to measure variables in a serial manner, which may have led to less accurate results. However, as this study was performed using a representative sample of the general South Korean population, and rigorous quality controls were applied to the study procedures in KNHANES, we believe that the data is valuable and should be taken into consideration. Also, since the latitude of South Korea ranges from 33 to 38 degrees north, all the people would not have been exposed to environments suitable for making adequate amounts of vitamin D from sunlight by themselves. Therefore, a geographic factor should also be taken as a meaningful adjusted variable. Moreover, by accounting for menopausal status, associations of serum ferritin with BMD would have been logically established.

In conclusion, increased serum ferritin levels were statistically significantly associated with a decrease in BMD of the lumbar spine in Korean premenopausal women, whereas no associations were found for serum ferritin with BMD of the total femur, femur neck and lumbar spine in women after menopause. This could be due to the fact that although increased serum ferritin is a well-documented change in postmenopausal women, there may be more comprehensive changes caused by menopause itself, which would affect BMD more profoundly.
